# Potentially inappropriate medication on communitydwelling older adults: Longitudinal analysis using the International Mobility in Aging Study

**DOI:** 10.7705/biomedica.6992

**Published:** 2024-05-30

**Authors:** Édison Pineda, Alejandra Fernández, Carmen Lucía Curcio, Juliana Fernandes de Souza, Afshin Vafaei, José Fernando Gómez

**Affiliations:** 1 Facultad de Ciencias para la Salud, Universidad de Caldas, Manizales, Colombia Universidad de Caldas Universidad de Caldas Manizales Manizales; 2 Grupo de Investigación en Gerontología y Geriatría, Facultad de Ciencias para la Salud, Universidad de Caldas, Manizales, Colombia Universidad de Caldas Universidad de Caldas Manizales Manizales; 3 Hospital Alma Mater de Antioquia, Medellín, Colombia Hospital Alma Mater de Antioquia Hospital Alma Mater de Antioquia Medellín Medellín; 4 Laboratório de Fisioterapia e Saúde Coletiva, Departamento de Fisioterapia da Universidade Federal de Pernambuco, Recife, Brasil Universidade Federal de Pernambuco Universidade Federal de Pernambuco Recife Brazil; 5 School of Health Studies, Western University, Kingston, ON, Canada Western University Western University Kingston Canada

**Keywords:** Potentially inappropriate medication list, polypharmacy, aged, independent living, prevalence, longitudinal studies, lista de medicamentos potencialmente inapropiados, polifarmacia, anciano, vida independiente, prevalencia, estudios longitudinales

## Abstract

**Introduction.:**

Medications are a fundamental part of the treatment of multiple pathologies. However, despite their benefits, some are considered potentially inappropriate medications for older people given their safety profile. Epidemiological data differences related to potentially inappropriate medications make it difficult to determine their effects on elderly people.

**Objective.:**

To estimate the prevalence and types of potentially inappropriate medications using the 2019 Beers Criteria® in a cohort of adults older than 65 years.

**Materials and methods.:**

We performed an observational, multicenter, retrospective, longitudinal study of a four-year follow-up of potentially inappropriate medications in community-dwelling older adults.

**Results.:**

We followed 820 participants from five cities for four years (2012-2016) and evaluated them in three different moments (m_1_ = 2012, m_2_ = 2014, and m_3_= 2016). The average age was 69.07 years, and 50.9% were women. The potentially inappropriate medication prevalence in the participants was 40.24%. The potentially inappropriate medications’ mean among the studied subjects in the first moment was 1.65 (SD = 0.963), in the second was 1.73 (SD = 1.032), and in the third was 1.62 (SD = 0.915). There were no statistical differences between measurements (Friedman test, value = 0.204). The most frequent potentially inappropriate medications categories were gastrointestinal (39.4%), analgesics (18.8%), delirium-related drugs (15.4%), benzodiazepines (15.2%), and cardiovascular (14.2%).

**Conclusions.:**

About half of the population of the community-dwelling older adults had prescriptions of potentially inappropriate medications in a sustained manner and without significant variability over time. Mainly potentially inappropriate medications were gastrointestinal and cardiovascular drugs, analgesics, delirium-related drugs, and benzodiazepines.

## Resumen

**Introducción.:**

Los fármacos son parte fundamental del tratamiento de múltiples enfermedades. Sin embargo, a pesar de sus beneficios, algunos se consideran medicamentos potencialmente inapropiados en adultos mayores, dado su perfil de seguridad. Las diferencias en los datos epidemiológicos relacionados con los medicamentos potencialmente inapropiados dificultan el establecimiento de sus efectos en adultos mayores.

**Objetivo.:**

Estimar la prevalencia longitudinal y los tipos de medicamentos potencialmente inapropiados, utilizando los criterios Beers® del 2019 en una cohorte de adultos mayores de 65 años.

**Materiales y métodos.:**

Se realizó un estudio observacional, multicéntrico, retrospectivo y longitudinal, de cuatro años de seguimiento de los medicamentos potencialmente inapropiados en adultos mayores de la comunidad.

**Resultados.:**

Se evaluaron 820 participantes de cinco ciudades durante cuatro años (2012 a 2016) en tres momentos (m_1_: 2012, m_2_: 2014 y m_3_; 2016). La edad promedio fue de 69,07 años y el 50,9 % eran mujeres. La prevalencia de medicamentos potencialmente inapropiados en los participantes fue del 40,24 %. El promedio de estos medicamentos entre los sujetos estudiados en el primer momento fue de 1,65 (DE = 0,963), en el segundo fue de 1,73 (DE = 1,032) y en el tercero fue de 1,62 (DE = 0,915). No hubo diferencias estadísticas entre las mediciones (prueba de Friedman, p = 0,204). Las categorías de los medicamentos potencialmente inapropiados más frecuentes fueron: gastrointestinales (39,4 %), analgésicos (18,8 %), relacionados con delirium (15,4 %), benzodiacepinas (15,2 %) y cardiovasculares (14,2 %).

**Conclusiones.:**

En cerca de la mitad de la población de adultos mayores de la comunidad, se prescribieron medicamentos potencialmente inapropiados de manera sostenida y sin variabilidad importante en el tiempo. Los más recetados fueron aquellos para tratar malestares gastrointestinales y cardiovasculares, analgésicos, para el delirium y benzodiacepinas.

The number of older people and life expectancy worldwide has significantly increased. It is estimated that the global geriatric population will grow at a rate close to 3% annually, a higher rate than any other age group ^1^. The significant increase in the elderly population in recent decades, especially in low- and middle-income countries, is a well-recognized situation. In 1990, the elderly population was approximately 6%, which increased to nearly 14% in 2020 and is expected to reach around 30% in 2050 ^2^. Aging is characterized by a progressive inability to maintain homeostatic balance and is associated with the decline of organ functions, which translates into a predisposition of the elderly population to develop multiple comorbidities ^3^.

In recent decades, the continuous use of medications has increased along with the number of non-communicable chronic diseases and life expectancy ^4^. Despite their benefits, some prescriptions are considered potentially inappropriate medications in older adults, meaning that medication risks of harm outweigh the potential benefits (i.e., not indicated or lacking evidence of efficacy, or medications not aligning with patients’ goals/preferences and values ^5^^,^^6^).

The Beers Criteria® have been used for identifying potentially inappropriate medications in multiple studies, and its most recent update was made in 2019 by the American Geriatrics Society (AGS) ^7^. Previous studies have investigated the risk of adverse reactions caused by potentially inappropriate medication in the treatment of chronic diseases, the safety of a single category of potentially inappropriate medication (anticholinergics) used by elderly patients, and the hazards of its use in elderly patients in continuous care ^8^. Results are variable, showing that potentially inappropriate medication prescriptions may be associated with potential risks for the elderly. However, this variability may be related to discordance of parameters such as prevalence given the variability in the methodology used in different studies (cross-sectional, longitudinal) or the elderly place of evaluation (institution, hospital general ward or intensive care unit, or community) ^9^^-^^15^.

Therefore, the purpose of this study was to determine the prevalence of potentially inappropriate medication in community-dwelling elderly individuals with sustained exposure to it and to characterize the most frequently prescribed potentially inappropriate medication groups.

## Materials and methods

### Universe

We conducted a longitudinal analysis using data from the International Mobility in Aging Study (IMIAS), a population-based study of 2002 communitydwelling older adults in five social and cultural contexts: Kingston (Ontario, Canada), Saint-Hyacinthe (Quebec, Canada), Tirana (Albania), Manizales (Colombia), and Natal (Brazil). The objective of this study was to understand how factors throughout life affect mobility in older adults. The characteristics and details of the study have been described elsewhere ^16^.

The present study followed the guidelines of the STROBE (Strengthening the Reporting of Observational Studies in Epidemiology) statement for reporting observational studies ^17^. For this study, we used data from older adults who were assessed in 2012 (m_1_), re-evaluated in 2014 (m_2_), and in 2016 (m_3_). The AGS 2019 Beers Criteria® ^7^ were operationalized into categories and recommendations pinpointing potentially inappropriate medications to identify older adults exposed to them. Participants considered for follow-up were older adults who had all their demographic and clinical data recorded in the database and who were taking potentially inappropriate medication, identified in moments m_1_, m_2_, and m_3_ (exposed). Additionally, for illustrative purposes, we conformed a group of older adults without exposure to potentially inappropriate medication at any of the evaluation times (m_1_, m_2_, and m_3_) (unexposed). The rest of the participants, those who presented potentially inappropriate medication in only one or two of the evaluation times, were excluded from the analysis.

### Data collection

The information was collected in the participants’ homes by trained interviewers and physicians using structured questionnaires in m_1_, m_2_, and m_3_. The questionnaire included information about the medications used daily by each participant. We recorded the data according to the protocol of the IMIAS study. Participants were interviewed and assessed after reading and signing the informed consent.

### Main measurement

We implemented operationalization of the AGS 2019 Beers Criteria® for the identification of potentially inappropriate medications ^7^. These criteria have been previously validated and have better performance for detecting potentially inappropriate medications in community-dwelling individuals ^18^^,^^19^. With the recorded information, we established 36 categories and 256 potentially inappropriate medication identifying recommendations. However, we had to exclude 40 recommendations due to the absence of data to stratify the renal function of the participants, resulting in 81.5% use of the recommendations. Subsequently, based on the 216 used recommendations, we designed and developed a specialized software, using the Synthax® programming language, to identify the presence or absence of potentially inappropriate medications in each of the 4,350 prescriptions (one per participant in each of the three evaluation moments: m_1_, m_2_, and m_3_) and to assign the category to each potentially inappropriate medication. We defined sustained exposure as one or more potentially inappropriate medications identified in all three evaluation times.

*Sociodemographic covariates.* We represented sociodemographic data with a dichotomous variable for gender, a discrete variable for age, and polytomous variables for marital status and recruitment city.

*Clinical variables.* The explored clinical variables were the number of chronic diseases, prescribed medications, and falls in the last year. The number of chronic diseases was estimated by summing up the pathologies examined in the structured questionnaire (hypertension, diabetes, cancer, chronic pulmonary disease, heart disease, stroke, osteoarthritis, and osteoporosis). The number of prescribed medications was obtained by reviewing all the formulas and recording the total number of drugs consumed daily by each participant. The number of falls in the last year was estimated by asking: “How many times have you fallen in the last 12 months?”. The use of health services was analyzed using the variable “number of visits to the doctor in the last year,” defined by the question “How many times have you seen a doctor in the last year?”.

### Statistical analysis

We performed a descriptive analysis (frequencies, distribution, means, and standard deviation). We conducted a cross-sectional bivariate analysis using the Student t test and Mann-Whitney U test according to the variables distribution, and *χ* ^2^ and Fisher exact test for categorical variables collected in m_1_. Data normality was measured using the Kolmogorov-Smirnov test. We ran a longitudinal bivariate analysis to estimate the proportion change of potentially inappropriate medication over the four years using Cochran’s Q test and Friedman test in participants exposed to potentially inappropriate medications. All statistical analyses were carried out using the IBM SPSS™ package, version 24.0, for MacOS (SPSS Inc., Chicago, IL, USA).

### Ethical considerations

We obtained approval from the ethics review committees of research centers at the University of Montreal Hospitals, Queen’s University (Kingston), the Institute of Public Health of Albania, the *Universidade Federal de Rio Grande do Norte* (Brazil), and the *Universidad de Caldas* (Colombia). Regarding Resolution 8430/1993 of the *Ministerio de Salud* of Colombia about research with human subjects, this study was considered to have minimal risk since the participants had a very low probability of suffering harm during the study.

## Results

**Figure 1:**
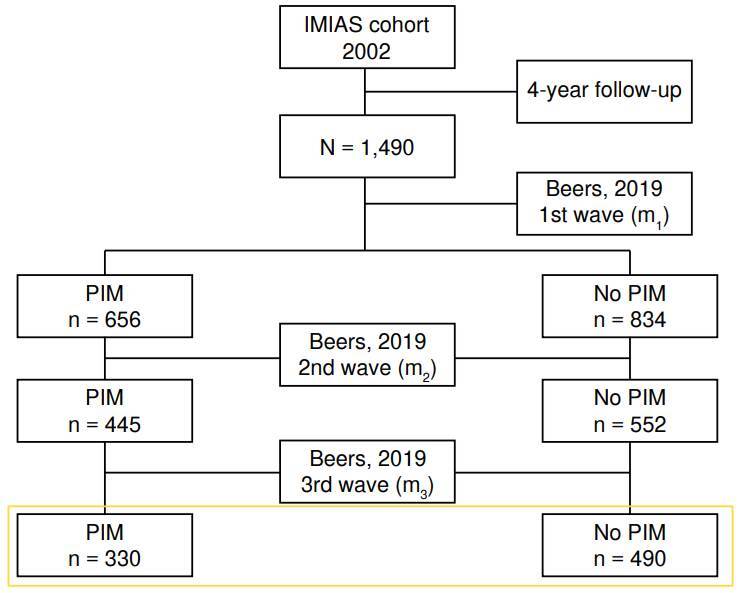
Participant selection

Out of the initial 2,002 participants, 25.57% (512) were considered lost to follow-up, resulting in 1,490 older adults to whom we applied the AGS 2019 Beers criteria in the m_1_, m_2_, and m_3_ moments. Among these, we excluded 610 individuals either because they had potentially inappropriate medication detected in only one or two of the three evaluation times, or because of the absence or potentially inappropriate medication in one or two of the three moments. The remaining participants were 820: 330 for analysis and 490 for comparison purposes (figure 1).

Table 1 presents the baseline characteristics of the study subjects, highlighting statistically significant differences between participants exposed and not exposed to potentially inappropriate medication. Just over half of the participants (50.9%) were women. However, the proportion of women between groups was higher in the exposed group (58.5% vs 45.7%; p = 0.000). The average age was 69.07 years.

**Table 1:** Characteristics of the study sample in 2012

Características		Total (N = 820)	Potentially inappropriate medication (n = 330)	No potentially inappropriate medication (n = 490)	p value
Women (n, %)*		417 (50.9)	193 (58.5)	224 (45.7)	0.000
Age (years, SD)		69.07 (2.85)	69.23 (2.97)	68.97 (2.78)	0.258
Marital status (n, %)					0.654
	Single	50 (6.1)	22 (6.7)	28 (5.7)	0.093
	Married	557 (67.9)	218 (66.1)	339 (69.2)	0.000
	Widowed	117 (14.3)	48 (14.5)	69 (14.1)	0.000
	Separated/Divorced	96 (11.7)	42 (12.7)	54 (11)	0.000
Highest level of education (n, %),					0.025
	Primary/illiterate	334 (40.7)	114 (34.5)	220 (44.9)	0.000
	Secondary	112 (13.7)	47 (14.2)	65 (13.3)	0.022
	Post-secondary	374 (45.4)	169 (51.2)	205 (41.8)	0.027
Recruitment site (n, %)					0.134
	Kingston	172 (21)	79 (23.9)	93 (19)	0.035
	Saint Hyacinthe	184 (22.4)	89 (27)	95 (19.4)	0.273
	Tirana	156 (19)	77 (23.3)	79 (16.1)	0.000
	Manizales	183 (22.3)	69 (20.9)	114 (23.3)	0.018
	Natal	125 (15.2)	16 (4.8)	109 (22.2)	
Number of chronic illnesses (mean, SD)*		1.81 (1.3)	2.3 (1.3)	1 (1.18)	
Number of prescribed medications (mean, SD)*		4.08 (2.99)	6.18 (2.87)	2.66 (2.12)	
Number of falls in the last year (mean, SD)*		0.27 (1.605)	0.67 (2.479)	0 (0)	
Number of visits to the doctor in the last year (mean, SD)*		4.77 (4.75)	6.13 (5.16)	3.86 (4.2)	
Self-reported health (n, % good health)*		524 (63.9)	197 (59.7)	327 (66.7)	
Quality of life (mean, SD)*		7.38 (2.21)	7.21 (2.16)	7.49 (2.24)	
Life-space (n, % not restricted)		640 (78)	251 (76.1)	389 (79.4)	
Depression (n, % without depression)*		668 (81.5)	258 (78.2)	410 (83.7)	
Cognitive function (n, % without cognitive impairment)		795 (97)	318 (96.4)	447 (97.3)	
SPPB (mean, SD)*		9.9 (1.97)	9.56 (2.1)	10.13 (1.82)	
Frailty in 2012 (n, % not frail)*		780 (95.1)	307 (93)	473 (96.5)	

SPPB: Short Physical Performance Battery; SD: standard deviation

* p value < 0.05 for χ^2^, Student t test or Mann-Whitney U test depending on the characteristics of each variable

Approximately 63.9% of study subjects reported their health as good, and when comparing exposure groups, the non-exposed reported their health as good more frequently than the exposed one (66.7% vs 59.7%; p = 0.022). Exposed older adults had, on average, more chronic diseases than the nonexposed group [2.3 (SD = 1.3) vs 1 (SD = 1.18); p = 0.000].

Overall, the average number of medications per formulation was 4.08 (SD = 2.99). By exposure group, the average was higher in the exposed than in the non-exposed [(6.18 (SD = 2.87) vs 2.66 (SD = 2.12); p = 0.000]. In the non-exposure group, no participants reported falls in the last year.

The mean number of doctor visits in the past year was higher in the exposure group than in the non-exposure group [6.13 (SD = 5.16) vs 3.86 (SD = 4.2); p = 0.000]. Non-exposed participants to potentially inappropriate medication had, on average, better quality of life than those exposed to potentially inappropriate medication [7.49 (SD = 2.24) vs 7.21 (SD = 2.16); p = 0.027].

The average score on the Short Physical Performance Battery (SPPB) test was lower in older adults exposed to potentially inappropriate medication than in the non-exposed group [9.56 (SD = 2.1) vs 10.13 (SD = 1.82); p = 0.000]. Of the participants exposed to potentially inappropriate medication, 7% presented frailty, twice the percentage of their non-exposed counterparts (p = 0.018).

In m_1_, 12 different categories of potentially inappropriate medications were used by older adults, with the most frequently consumed being gastrointestinal (39.4%), analgesics (18.8%), delirium-related drugs (15.4%), benzodiazepines (15.2%), and cardiovascular (14.2%). Four years later (m_3_), 11 categories of potentially inappropriate medications were identified, the following as the most common: gastrointestinal (43.1%), analgesics (14.8%), cardiovascular (14.5%), delirium-related drugs (14.2%), and benzodiazepines (13.1%). About half of exposed participants received at least one potentially inappropriate medication (prevalence = 40.24%). The average of potentially inappropriate medications among exposed subjects in m_1_ was 1.65 (SD = 0.963), in m_2_ was 1.73 (SD = 1.032), and in m_3_ was 1.62 (SD = 0.915), with no statistically significant differences between measurements (Friedman test, p value = 0.204).

**Figure 2:**
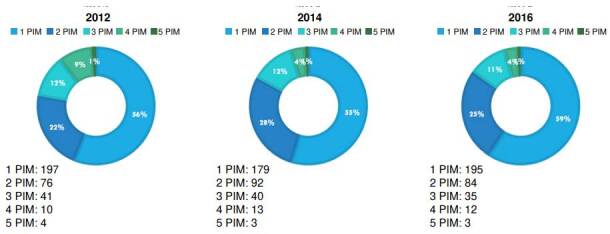
Distribution of the number of potentially inappropriate medications per person per year

Table 2 shows the number of potentially inappropriate medications used per person during the 4-year follow-up, with no statistically significant differences in the distribution of those medications during the three evaluation periods, and the results were not modified when adjusted for the different covariates (figure 2).

**Table 2:** Number of potentially inappropriate medication per person

Variable	m_1_ (2012) (%)	m_2_ (2014) (%)	m_3_ (2016) (%)	p value *
1 Potentially inappropriate medication	56.4	54.6	59.1	0.291
2 Potentially inappropriate medications	22.3	27.8	25.4	0.151
3 Potentially inappropriate medications	12.4	12.1	10.6	0.382
4 Potentially inappropriate medications	8.9	3.9	3.6	0.094
5 Potentially inappropriate medications	1.2	0.9	0.9	0.073

m_1_: moment 1; m_2_: moment 2; m_3_: moment 3

* Cochran's Q

## Discussion

We conducted a longitudinal analysis of sustained exposure to potentially inappropriate medications, establishing their prevalence, distribution, and classification in a community-dwelling population of older adults. During the study period, from 2012 to 2016, approximately half of the participating older adults received at least one potentially inappropriate medication, consistent with estimates made using different versions of the AGS Beers criteria ^9^^-^^12^. Chinthalapudi *et al*. reported a prevalence of potentially inappropriate medication using Beers 2019 criteria of 54% in a third-level center ^10^. Recently, a longitudinal study including participants from a US center that used 2019 Beers criteria found a prevalence of this medication of 34.4%. Furthermore, there was a significant decrease in the prevalence, from 35.3% in 2011 to 32.5% in 2015 ^13^.

In the present analysis, we did not find a proportion change of potentially inappropriate medication per participant in the four-year follow-up, explained maybe by the presence of participants from various cities with different modifying factors of potentially inappropriate medication exposure, contributing to the homogenization of the global sample. We confirmed this by reviewing the results of the population-based study by Roux *et al*., who applied 2015 Beers criteria in a one-year follow-up study and showed that 25.1% of potentially inappropriate medication users, prescribed at the beginning, continued to use them one year later. The risk of persistence with this type of medication increased by 10% for the most socially deprived individuals (RR = 1.10, 95% CI = 1.05-1.15). There was no significant difference between the different quintiles of the material deprivation index (except for the first quintile, least deprived individuals) ^14^. The high frequency observed may have been influenced by new medications included in the Beers 2019 list and the classification of potentially inappropriate medications in this study based on the amount.

Additionally, this approach may have influenced the stability in the prevalence of this type of medication used over time. Among participants who had sustained exposure to this type of medication, just over half received one drug, nearly a quarter received two, and a little over 10% received three potentially inappropriate medications at each of the three-time points analyzed.

These proportions are consistent with those reported in a populationbased study from Malaga conducted by Blanco-Reina *et al*., who indicated a distribution of 46.2% for one potentially inappropriate medication, 28.5% for two, and 13.7% for three, according to 2015 Beers criteria® ^12^. The categories most frequently used by participants were gastrointestinal, analgesics, delirium-related drugs, benzodiazepines, and cardiovascular drugs, which are comparable to those described by Moriarty *et al*. in 2020. They indicated a high usage of proton pump inhibitors, strong anticholinergics, benzodiazepines, and non-steroidal anti-inflammatory drugs. These medications were identified using the 2012 Beers criteria ^20^.

In a literature review, we did not find other studies using potentially inappropriate medication categories to characterize the use of these medications, probably due to the difficulties in grouping those associated with different risks, such as anticholinergics linked to delirium, risk of falls, and cognitive impairment ^20^^-^^22^.

One strength of this study is the use of an international database with detailed sociodemographic and clinical characteristics of the participants and their four-year follow-up. Additionally, the presence of culturally diverse cities from middle- and high-income countries allowed the study of the relationship between potentially inappropriate medication and the health of older adults, which may be considered more representative of the global population compared to single-center or single-country studies. Another strength is the use of standardized tools in the five cities of the IMIAS study, which reduces data collection variability. Moreover, specialized software used to identify potentially inappropriate medications reduces errors in quantification and characterization.

We identify several limitations like the inability to detect interruptions in potentially inappropriate medication use over four years or to determine other potentially inappropriate medications added to participants’ treatment. Some recorded variables were self-reported, and there were no other sources to validate the information. It could result in underreporting due to poor recall, which may differ between cities. Finally, analysis of medications requiring dose adjustment based on renal function using 2019 Beers criteria will be necessary.

In conclusion, sustained use of potentially inappropriate medications was present in more than half of the older adult population in the community. The most frequent medications included gastrointestinal, analgesics, delirium- related drugs, benzodiazepines, and cardiovascular, with little variability over time. Reducing potentially inappropriate medication use is part of the World Health Organization’s global health agenda and has been a priority since 2017, which proposed reducing potential risks associated with medication use ^23^.

The results reinforce the need to intervene in the medication prescribing process to prevent the continued formulation of inappropriate and unnecessary medications for older adults ^24^^-^^26^. Additionally, the findings of this study will help identify particularly vulnerable individuals who need prevention and deprescribing strategies.
